# An explainable machine learning framework for lung cancer hospital length of stay prediction

**DOI:** 10.1038/s41598-021-04608-7

**Published:** 2022-01-12

**Authors:** Belal Alsinglawi, Osama Alshari, Mohammed Alorjani, Omar Mubin, Fady Alnajjar, Mauricio Novoa, Omar Darwish

**Affiliations:** 1 School of Computer, Data and Mathematical Sciences, Western Sydney University, Rydalmere, 2116 NSW Australia; 2 Oncology Division, Department of Internal Medicine, Faculty of Medicine, Jordan University of Science and Technology, Irbid, Jordan; 3 Department of Pathology and Microbiology, Faculty of Medicine, Jordan University of Science and Technology, Irbid, Jordan; 4 College of Information Technology, UAE University, Al-Ain, UAE; 5 The School of Engineering, Design and Built Environment, Western Sydney University, Rydalmere, 2116 NSW Australia; 6 Department of Information Security and Applied Computing, Eastern Michigan University, Michigan, 48197 USA

**Keywords:** Lung cancer, Outcomes research

## Abstract

This work introduces a predictive Length of Stay (LOS) framework for lung cancer patients using machine learning (ML) models. The framework proposed to deal with imbalanced datasets for classification-based approaches using electronic healthcare records (EHR). We have utilized supervised ML methods to predict lung cancer inpatients LOS during ICU hospitalization using the MIMIC-III dataset. Random Forest (RF) Model outperformed other models and achieved predicted results during the three framework phases. With clinical significance features selection, over-sampling methods (SMOTE and ADASYN) achieved the highest AUC results (98% with CI 95%: 95.3–100%, and 100% respectively). The combination of Over-sampling and under-sampling achieved the second-highest AUC results (98%, with CI 95%: 95.3–100%, and 97%, CI 95%: 93.7–100% SMOTE-Tomek, and SMOTE-ENN respectively). Under-sampling methods reported the least important AUC results (50%, with CI 95%: 40.2–59.8%) for both (ENN and Tomek- Links). Using ML explainable technique called SHAP, we explained the outcome of the predictive model (RF) with SMOTE class balancing technique to understand the most significant clinical features that contributed to predicting lung cancer LOS with the RF model. Our promising framework allows us to employ ML techniques in-hospital clinical information systems to predict lung cancer admissions into ICU.

## Introduction

Managing hospital bed availability and efficiency is obligatory for addressing challenges associated with the overabundance of patients in ICU and hospital and avoiding ICU beds shortage, especially in uncertainties such as pandemics^1^. Most significantly, the key aspects are minimizing the risk associated with acquired infection during ICU hospitalization, mortality risk^2^, and medical complications for vulnerable patients. Improved hospital resources and planning have the potential to mitigate and minimize these risks^3,4^. Therefore, a lower ICU Length of Stay (LOS) than necessary is associated with lower total hospital charges. Consequently, hospital resources are well-managed, and better outcomes are achieved for the patients^5^.

Traditional LOS calculation methods are currently in use, such as ICU APACHE versions (I, II, III, IV), SAPS^6–9^, and SOFA^10^. These methods use patients’ features or ICU features to estimate the inpatient LOS during hospital admission. However, they suffer from poor performance as they are not disease-specific prediction methods. Further, there is consensus on the most suitable methods for ICU LOS^11^. Most hospital managements use electronic healthcare records (EHR) to facilitate their daily operational and medical procedures and LOS determination. The EHR healthcare assessment systems store data associated with patients’ encounters, such as their demographics, diagnosis, laboratory tests, prescriptions, radiological images, clinical notes, and many more^12,13^.

Machine learning algorithms have been used in medical imaging and genomics; however, their use to model clinical outcomes is less well-established^14^. Nevertheless, whether in the ICU or otherwise, Hospital LOS is one of such important outcomes, whose prediction relies on such techniques as per recent literature. Moreover, these techniques are broadly generalizable, and scientists can build ensembles based on these algorithms to predict many other clinical outcomes.

In the literature, state-of-the-art ML models (ensemble methods)^15,16^ were studied in the context of emergency department LOS prediction. Multivariate- ate analysis-based studies^17,18^ are examined in the ICU predicting LOS within the ICU context. Recent attempts applied by Deep learning-based regression techniques such as Bayesian Neural Network (BNN)^19^, Short Long Term Memory (LSTM) for time-series prediction^18^. In many studies, predicting LOS with regression-based predictive models is studied extensively^11,20–23^. At the same time, most of these studies are focused on emergency departments (ED) or cardiovascular-related admission to ICU units or patients who stayed in ICU after the surgical or medical intervention using classification approaches such as those indicated in this study^24^.

A limited number of cancer-based studies assessed the predictive models in the context of lung cancer LOS from EHR and data-driven using machine learning algorithms. For instance, Best et al.^25^ evaluated multivariate regression with Spearman correlation as the features selection method to predict inpatients length of stay complications after lobectomy for lung cancer at three different treatment healthcare facilities. Similar research by Li et al.^11^ applied the multivariate logistic regression with a manual features selection way to predict the effects of pulmonary fissure completeness on postoperative cardiopulmonary complications and hospital length of stay in patients for early-stage non-small-cell lung cancer. Logistic regression-based studies are another favourable approach. For example, Dong et al.^26^ analyzed the effectiveness of oxygen desaturation (EOD) and heart rate to predict major postoperative cardiopulmonary complications for non-small cell lung cancer patients using binary logistic regression. Comparable research by Pompili et al.^27^ examined the logistic regression model to assess whether the quality of life (QoL) scales are associated with the prolonged LOS postoperative hospital stay for lung cancer patients and enhancement of patient healthcare quality after surgery recovery. Although these approaches obtained good predicted outcomes using the regression or logistics based results, their work did not evaluate the power of predictive models in decision-making to facilitate the workload in-hospital healthcare and the management of hospitals and healthcare structures. Moreover, the literature did not report a comprehensive work that considered benchmarking and comparing models. We did not observe a data-driven machine learning approach treating the imbalance class common problem in the predictive classifier. There were no attempts to utilize the advancement of explainable artificial intelligence (xAI) methods that aimed to explain the decision-making and working inners of machine learning models for better understanding of the data-driven insights and, therefore, improving the hospitals booking of facilities and resources utilization.

Lung cancer patients attribute roughly 27% of solid cancer ICU admissions^28,29^. Lung cancer patients are perceived to receive substantially worse ICU outcomes compared to other cancer types. On an additional note, most lung cancer-based studies reported descriptive statistics about the hospitalization characteristics such as the median or mean and *p*-Value^30^. Predicting LOS cancer-based studies^24,28^ are less prevalent in the literature review within the context of inpatient admission to ICU. LOS lung cancer-based machine learning studies with a classification-based focus are scarce. We have not observed ML studies that examined the LOS predictive models for lung cancer ICU hospitalizations to the best of our knowledge. Furthermore, we have not found relevant studies that examined class-balancing methods with ML techniques to predict cancer LOS tasks, especially cancer-based studies in the ICU healthcare context. This study aims to develop a predictive machine learning research framework to predict lung cancer inpatients length of stay at the time of ICU admission based on the data fed to the ML models from the electronic hospital medical records.

Our research motivation takes the opportunity to explore the LOS prediction as a health assessment metric for resource utilization in the ICU settings with ML classification approaches.

Our key contributions in this study are to Introduce a doable data-driven framework to predict the Length of Stay for unexplored research topic (lung cancer) admitted patients to the ICU. The study provides a practical framework to deal with an imbalanced classification problem in EHR datasets. Hence, the problem is deterministic for machine learning models’ performance in healthcare analytics, particularly electronic medical records. The research framework will examine the problem using different six class-balancing algorithms. Thereafter, our proposed framework deliberates the EHR data’s dimensionality hardened issue by focusing on clinically significant attributes (Lung Cancer diagnosis) as input features. Furthermore, we utilize the features selection method Recursive features elimination (RFE) in the Lung Cancer LOS to eliminate the worst-performing features and select the subset of features associated with the target predicted LOS class. Thus, the optimal features selection method will be evaluated further against the six class-balancing methods to achieve the desired predicted outcomes of LOS lung cancer. Finally, our new predictive approach utilizes the explainable machine learning approach (SHAP) that fits the outperforming classifier with the clinically appropriate class balancing method in the context of binary class prediction problems.

## Results

**Figure 1:**
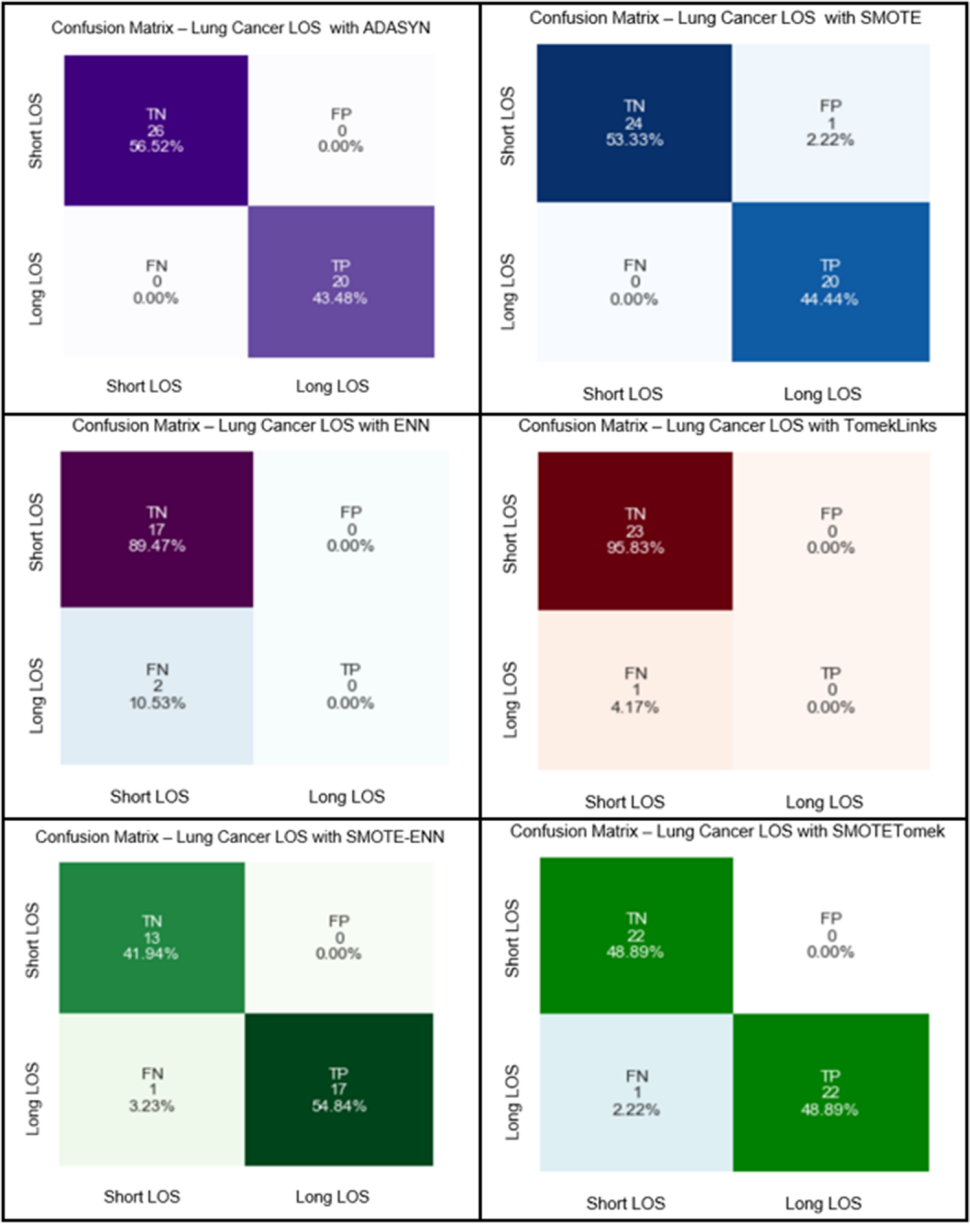
Confusion matrix for class-balancing techniques for lung cancer LOS with CS.

We exploited various class-balancing methods in the second phase to compare the RF model performance on each technique. We fitted the six class balancing methods into the RF classifier and evaluated their performance (AUC). We used the clinical significance (CS) with all features (75 features) in the lung cancer subset. We have also exploited the selection procedure RFE with the (Top 60 (Supplementary file: S8.3, Fig. 6) of the RF model-based features selection technique to predict the short and long Length of Stay with imbalanced data. The RF model successfully predicted the Short LOS and the Long LOS with the highest reported predicted IBA score (100%) and (100% for Sensitivity and specificity) using the class-balancing Over-sampling technique (ADASYN) in both CS and RFE subsets. SMOTE obtained IBA score of (96%) with (98% sensitivity and specificity) as the second most desired result in class-balancing with the Over-sampling approach (for CS and RFE) subset. Under-Sampling methods followed an opposite trend, while they attained a 0% IBA score for ENN and TomekLinks, respectively following (CS and RFE) in the feature selection procedures. Moreover, the specificity dropped down drastically, and both class-balancing methods reached the lowest specificity scores (11% and 4%) correspondingly in both approaches (CS and RFE) (Fig. 3). The combination of over-and under-sampling methods (SMOTETomek and SMOTE-ENN) reported the same results in the CS and RFE approaches. SMOTETomek gained high desired predicted outcomes with 96% IBA score and (98% for Sensitivity, and specificity) evaluation metrics. The SMOTE- ENN showed compacted results for both classes (Short and Long LOS) with (94%: IBA, 97% sensitivity, and 98% specificity) respectively. We exploited the confusion matrix for further evaluation and described the RF classification model’s predictive outcomes on the testing dataset. In (Fig. 1), TP is donating the percentage of the number of samples that have been correctly predicted. The TN refers to the percentage of the outcomes where the RF correctly predicted the true negative (Short LOS/Long LOS). In contrast, FP denotes the percentage of the RF predicted outcomes.

The model incorrectly predicted the positive class, and the FN is the outcome when the RF model incorrectly predicted the negative class (Short LOS/Long LOS). As seen (Supplementary file: Fig. 8), the Over-sampling method (ADASYN) successfully predicted the TN and TP (56.52 and 43.48%), respectively, for the (Short LOS and Long LOS) classes. Further, ADASYN did not commit any false predictions (FP or FN). Similarly, SMOTE (Over-sampling ) efficaciously predicted (Short/Long) LOS classes with (TP: 44.44% and TN: 53.33%). The RF-SMOTE rate in the FP was minimal, as well for (FN = 0%). Nonetheless, the Under-sampling class-balancing methods reported unreliable predicted results with TN (89.47% and 95.83%) for ENN and Tomeklinks, respectively. TP percentages for both class balancing methods were very poor (0%) for each of them. On the other hand, the Combination with Over/Under-sampling methods such as SMOTE-ENN and SMOTE-Tomek revealed desired results and that TN and TP rate was equal (48.89%) in the SMOTE-Tomek and (41.94%-54.84%) in the SMOTE- ENN. Both techniques reported rates for FN with (1 prediction) for both models and (0%) in the case of FP. All reported results are discussed in Table 1.

**Table 1:** A Comparison between class-balancing methods using clinical significance features selection (CS) and random forest model with (*Confidence Interval “CI %95”).

Method	Sensitivity*	Specificity	AUC*	IBA*	G.mean*
SMOTE-CS	98% [95.3-100]	98% [95.3–100]	98%[95.3–100]	96% [92.2–99.8]	98% [95.3–100]
ADASYN-CS	100%	100%	100%	100%	100%
ENN-CS	89% [82.9–95.1]	11% [4.9–17.1]	50% [40.2–59.8]	0%	0%
TomekLinks-CS	96% [92.2-99.8]	4% [0.2–7.8]	50% [40.2]	0%	0%
SMOTETomek-CS	98% [95.3–100]	98% [95.3–100]	98% [95.3–100]	96% [95.3–100]	98% [95.3–100]
SMOTE-ENN-CS	97% [93.7–100]	98% [95.3–100]	97% [93.7-100]	94% [89.3–98.7]	97% [93.7–100]

## Discussion

The present study demonstrates machine learning algorithms’ application to predict the ICU Length of Stay for lung cancer patients. In our study (Supplementary: Sects. S8.1, S8.2 and S8.3), we compared the RF against the XGBoost and Logistic Regression (LR) in the baselining stage. The RF showed a better performance in both clinical features selection and RFE. Therefore, it was the winning model to apply class-balancing using the six methods in our study. This experiment aimed to examine the model’s selection first in the baselining stage with cross-validation. Then, the outperforming model is further evaluated based on the study motivation. This applies to any classifier of the bed’s manager selection or the clinician to compare the predictive performance of more sets of classifiers in a more comprehensive practical application. We anticipate our previous work^31^ that utilized a set of classifiers with more models comparisons and the other published work in the literature, such as^32^ which followed a similar approach.

Our study reports several important findings. We benchmarked three classifiers (Random Forest, XGBoostand Logistic Regression) using the cross-validation method (k-fold = 10). We observed from the reported results XGBoost classifier’s mean re- ported accuracy performance fluctuated among different RFE top feature sets and CS feature selection methods. Nevertheless, logistic regression was the fastest model to train, whereas the RF and XGBoost both models needed more time to solve their prediction outcomes. The RF is the most computational costly model among all three of them. Additionally, the Random Forest showed resistance to any changes in the features selection varieties such as the CS and RFE with the various top features approaches. Unlike other models, RF improved by adding more features in each (RFE) feature selection performance attesting with cross-validation (k-fold = 10). Thus, RF showed robust performance and reported stable results with different feature selection methods. We nominated RF as the winning model for the class-balancing stage. We tested and evaluated the six class-balancing methods with RF classifier. We aimed to examine the most robust class-balancing approaches (Over-sampling, Under-sampling, or the combination of both). We decided to report the AUC to measure the quality of the model’s prediction in each class balancing method (how well the RF model can distinguish between LOS classes “Short vs Long”). We have observed that CS outcomes (AUC scores for six class-balancing methods) are the same as RFE. Hence, we are referring to CS approach when discussing the reported results with class-balancing (AUC) performance measures. Over-sampling reported the best AUC scores (100% and 98%) for ADASYN and SMOTE. Correspondingly, the combination of both “SMOTETomek” and “SMOTE-ENN” came up as the second-best approach with 98% and 97%, respectively. Unlike the Over-sampling or the combination approach, the Under-sampling presented the weakest AUC results (50%) for both TomekLinks and ENN. It indicated that the two methods could not differentiate between the two classes (short LOS and long LOS), resulting in lower and undesired performance. Subsequently, under-sampling methods are not suitable for predicting inpatients’ Length of Stay. Eventually, we assuredly disregarded the TomekLinks and ENN from LOS predictions in binary class problems. The Over-sampling and Combination of (Over-sampling and Under-sampling) presented high predicted AUC results for the Short LOS and the Long LOS.

Both class-balancing approaches are considered for further clinical explanation to evaluate their clinical insights with the clinical oncologist. Further, to assess their feasibility and the clinical insights they may induce for utilizing hospital resources and hospital healthcare as- assessment systems in the ICU. The class balancing technique (ADASYN) reported the most successful predicted outcomes from the confusion matrix Fig. 1 on the test dataset. ADASYN distinguished distinctively two classes (Short LOS and Long LOS), where the RF did not report any false positive or false negative predictions. The second most crucial result concerning the class balancing methods came from (SMOTE), which showed the RF desired ability to efficiently differentiate between the two classes with only one minor false-positive prediction. Under-sampling class methods (ENN and TomekLinks) produced weak predictive outcomes and unreliable performance where both techniques provided high true negative ratios and zero outcomes for the true positive. In addition, both methods reported noticeable false negatives for how RF incorrectly predicts the positive class following both approaches. The (SMOTE- ENN and SMOTE-Tomek) are Combined between class under/over-sampling techniques, whereas their testified outcomes (true-positive and false-negative) are desired with minor incorrect predictions.

In practical application, the proposed methodological framework can be pipelined to achieve optimal predictive performance by allowing clinicians and beds’ managers to affirm the outperforming post-features selection method against the model choice. Recent research^33^ evaluated the usefulness of post-feature selection to obtain the desired predictive performance in hospital settings.

### Explanations with class-balancing using SHAP

**Figure 2:**
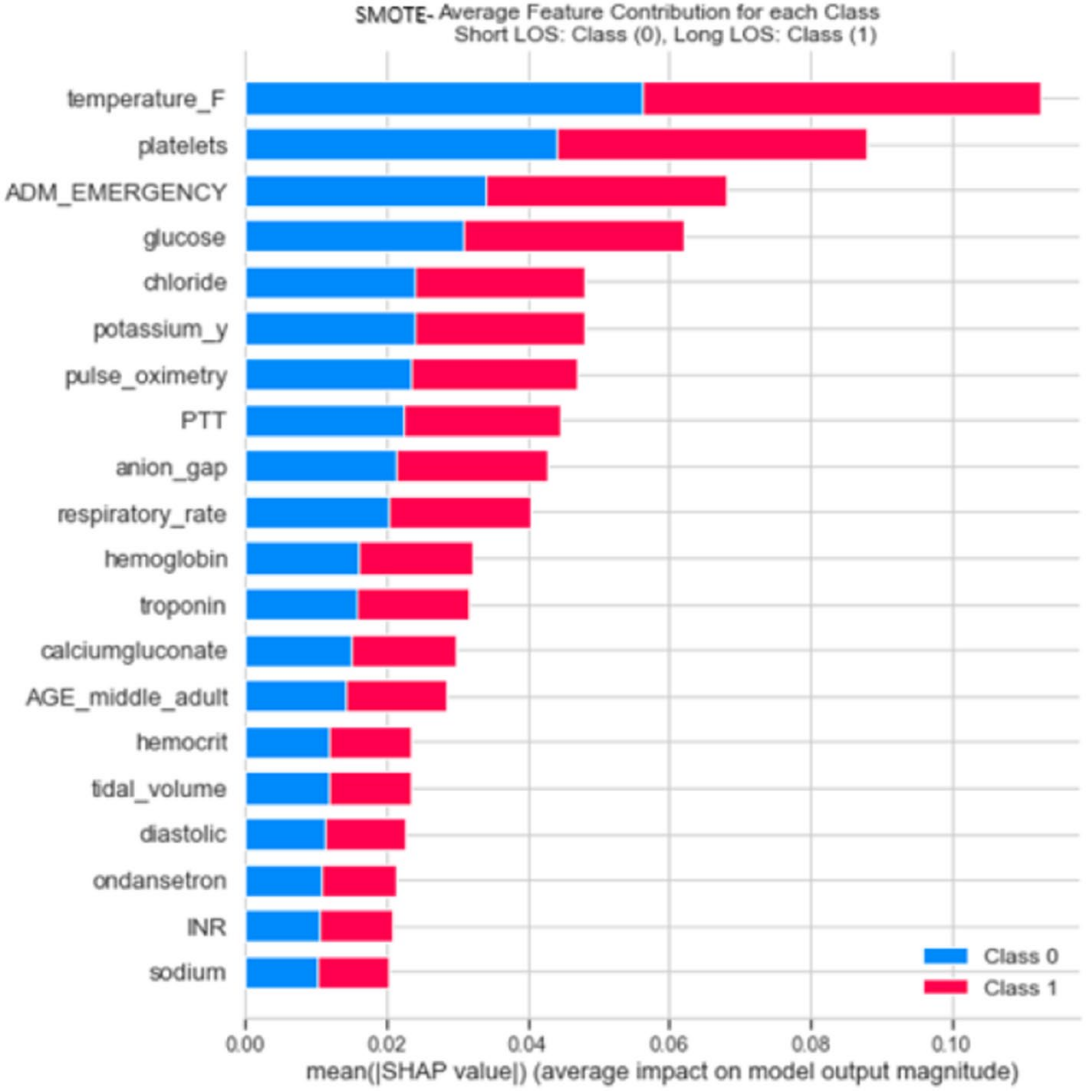
SHAP (mean value; the impact of each model’s (features) on the model output magnitude for selected Class-balancing methods with RF.

In a healthcare context, particularly in hospital clinical decisions or healthcare assessment systems, the Length of Stay continuum is important in decision-making^34^. The local explanation approach determines what variables (lung cancer features) explain the Random Forest’s specific prediction (LOS: short or long) using the class balancing methods as seen in Fig. 2. The Random Forest’s SHAP explanations (S8.5) for the dependable class balancing algorithms (SMOTE, ADASYN, SMOTEENN, and SMOTETomek) are depicted in (Supplementary file: Fig. 8). Top features such temperature (F), Emergency Admission “ADM_EMERGENT”, Glucose, respiratory rate (respiratory_rate)) were highly explained and are the most highly ranked features. This confirms how the SHAP using RF could rank clinical variables based on features’ importance with clinical soundness. While RF SHAP (SMOTE ENN ) ranked (systolic) variable in the top features, the diastolic came in the least in features by importance in the list. In terms of the SHAP (RF) model explainability for the class balancing techniques (Supplementary file: Fig. 8), we have observed that the SHAP explanation of the SMOTE is more definitive in the real practice and contented for clinicians.

Therefore, by using the SHAP ranking (mean SHAP value) in this study, we can judge that the sequence of data for (SMOTE and RF classifier features by importance) is more reliably related to the situation of the patients. For instance, patients sharing common demographic, diagnostic and laboratory features are supposed to require similar resource utilization; therefore, the SMOTE is expected to be efficiently able to quantify and standardize resource utilization for patients during their hospital stay. Further, we can achieve the provision for the LOS for the patients with better accuracy based on the SMOTE ranking of the lung cancer clinical variables. Thus, this verifies the RF model’s suitability and SMOTE reasonability for lung cancer LOS prediction in ICU.

### Research implication

Lung cancer is one of the common and most serious cancers, which in addition to being a cancer, it affects a vital organ, i.e. the lung, and may cause impairment of the function of the cardiopulmonary system. Moreover, the possible spread or paraneoplastic syndromes associated with some stages or types of lung cancer may act as players in the deterioration of the patient condition requiring further hospital ICU care. Listed here are some examples of such clinical features: the presence of lung infection, pleural effusion or pulmonary embolism, site of the tumor (e.g. tumor compressing the main bronchus), presence of recurrent laryngeal nerve paralysis causing hoarseness of voice and aspiration in the lungs may lead to increasing the LOS of patients admitted to the ICU. Other scenarios include superior vena cava syndrome when the cancer compresses the superior vena cava causing decreased oxygenation and fluid retention in the upper part of the patient’s chest, or when there is massive pericardial effusion or heart failure; all of these scenarios necessitate longer ICU stay. Moreover, the presence of brain metastasis may lead to an impaired level of consciousness and seizures, eventually increasing the length of the stay in the ICU.

Modelling LOS was chosen in this study because it is a primary reason for increasing cost. Accurate Modelling of this outcome can help healthcare systems identify risk factors for unnecessary hospital days of stay, potentially reduce waste, provide more efficient allocation of medical resources, and improve patient health care. Such models could be used to build an application into the background of EHRs to determine predicted outcomes automatically. The model provides patients and families with information to plan for work absences or care about discharge. Moreover, non-clinicians can utilize the predictions. For example, beds’ managers could ensure that adequate numbers of beds are available in intensive care units.

The lung cancer LOS prediction framework has several clinical research applications. Firstly, it can help the clinicians decide when to intervene with certain procedures or actions based on the SMOTE-RF scale (prediction), such as evaluating the patients’ severity at admission to determine the LOS. Secondly, the framework helps early clinical patient problems and critical situations requiring urgent intervention to accelerate medical decisions and treatments. Thirdly, it predicts the clinical course (anticipation) during the ICU admission. Last but not least, the framework helps the junior doctors practising in the ICU to manage the patients’ hospitalization based on the superior performance based on the methodological and experimented classifier (RF) and the appropriate class balancing method (SMOTE).

### Work limitations

Although our study has many advantages, it also has some limitations to address. We did not perform an external validation for the proposed framework on another medical dataset with similar characteristics to our study due to the lack of accessibility to other hospital data. This prevented us from verifying our predictive framework on real-world hospital data and attesting to the class balancing technique performance, especially the SMOTE. We are planning in our future work to validate our proposed predictive framework (LOS lung cancer) on a real hospital dataset that has similar attributes and characteristics as we examined in our current study.

Secondly, in our study, the sample size is relatively small due to the limited availability of the lung cancer diagnosis in the MIMIC-III 1.4 version dataset. This prevented us from evaluating the proposed models on a larger dataset for the same lung cancer diagnosis. Consequently, this did not exploit the advancement for deep learning techniques to predict lung cancer’s LOS and find further clinical insights or associations between the clinical variables in the disease-centred approach using deep neural networks. Furthermore, we did not perform the hyperparameter tuning procedure due to the limitation of the small dataset (lung cancer LOS subset). This decision was made to avoid the risk of overfitting.

Although the study assessed and examined the importance of predicting the LOS lung cancer predictions from ICU- hospitalizations, the authors refer to the significance of evaluating the predicted outcomes from enough hospitalized lung cancer cases for that eventually they are needed from statistically evaluation to provide robust predictive outcomes. Therefore, we courage researchers in the domain of hospital healthcare assessment for cancer-based studies in ICU settings to verify the machine learning models’ performance on a sufficient number of hospitalized cases to achieve robust results for LOS predictive tasks in real settings.

Finally, we did not study the relative accuracy of the model compared to clinician estimates of LOS. Still, we believe it can be used as a guide for quality improvement initiatives. For example, LOS indices which compare expected to observed LOS, have been proposed as efficiency and hospital performance markers. Using patient-specific predicted LOS to measure expected LOS may improve the accuracy of such indices, allowing hospitals to generate more representative quality metrics and, in reimbursement schemes that incentivize quality care, avoid punishment for taking on higher-risk patients.

## Methods

### Settings and data extraction

We conducted the study using the “Medical Information Mart for Intensive Care dataset (MIMIC-III v1.4). The MIMIC-III dataset compromises de-identified health-related data associated with adults patients (*N* = 53,423) who stayed in ICU between 2001 and 2012 at the Harvard Medical School’s teaching hospital (BIDMC) in Massachusetts, USA^35^. The MIMIC-III is a relational database consisting of data tables relating to patients who stayed at the ICU BIDMC hospital. The patients’ information such as demographic age, patient’s vital signs, laboratory and test results, medications, health, and medical procedures are linked by unique admission ID (HADMI D) amongst all database tables (EHR). The dataset has great advantages; (1) it is freely available for researchers worldwide. (2) it contains a diverse and substantial population of ICU patients. (3) it comprises high temporal resolution data such as electronic documentation, laboratory results, bedside monitor trends, and waveforms. Data characteristics associated with the lung cancer patients and the inclusion protocol for lung cancer patients from MIMIC-III is available from Table 1S2 in [Supplementary file: S2.1]. We obtained access to the database by taking an online course at the National Institutes of Health and passing the ’protection Human Research Participants’ exam (no. 8152360). The establishment of the MIMIC III database was approved by the institutional review board of Beth Israel deacons Medical Center and Massachusetts Institute of Technology. Informed consent was not required because all protected health information has been de-identified. All methods were performed in accordance with the relevant guidelines and regulations.

The LOS distribution was 85.58% for the Short LOS and 14.42% for Long LOS. The majority of the admitted cases of the population are senior adults, 80% (aged 65$+$) based on the inclusion criteria. Adults (Middle age: (> 35 years old & < 65) cases were 20%, and there were observations in the MIMIC-III dataset for the young age category (> 14 years old & < 36), and similarly no observations were available from the dataset for children age category (< 14 years old). The data showed that the population of admitted cases were (65.72%: male) and (34.73%: female).

### Data preprocessing and feature selection

Data pre-processing is deemed an essential task in the data mining process. Generally, datasets suffer from missing values, outliers, or raw data that require further processing and features redundancy^36^. Therefore, we performed several steps to process and extract (Lung Cancer LOS) before this work’s prediction stage.

- Inclusion Protocol for Lung Cancer Patients from MIMIC-III dataset [Supplementary file: S3.1]
- Feature Extraction, Missing Data Handling Data Imputation [Supplementary file: S2.1, S4.1].
- Discretizating Target Class (LOS): the Short LOS (0–7 days) and the Long LOS to (>7 days) [Supplementary file: S4.2]
- Categorical Variable Transformation [Supplementary file: S4.3].

**Figure 3:**
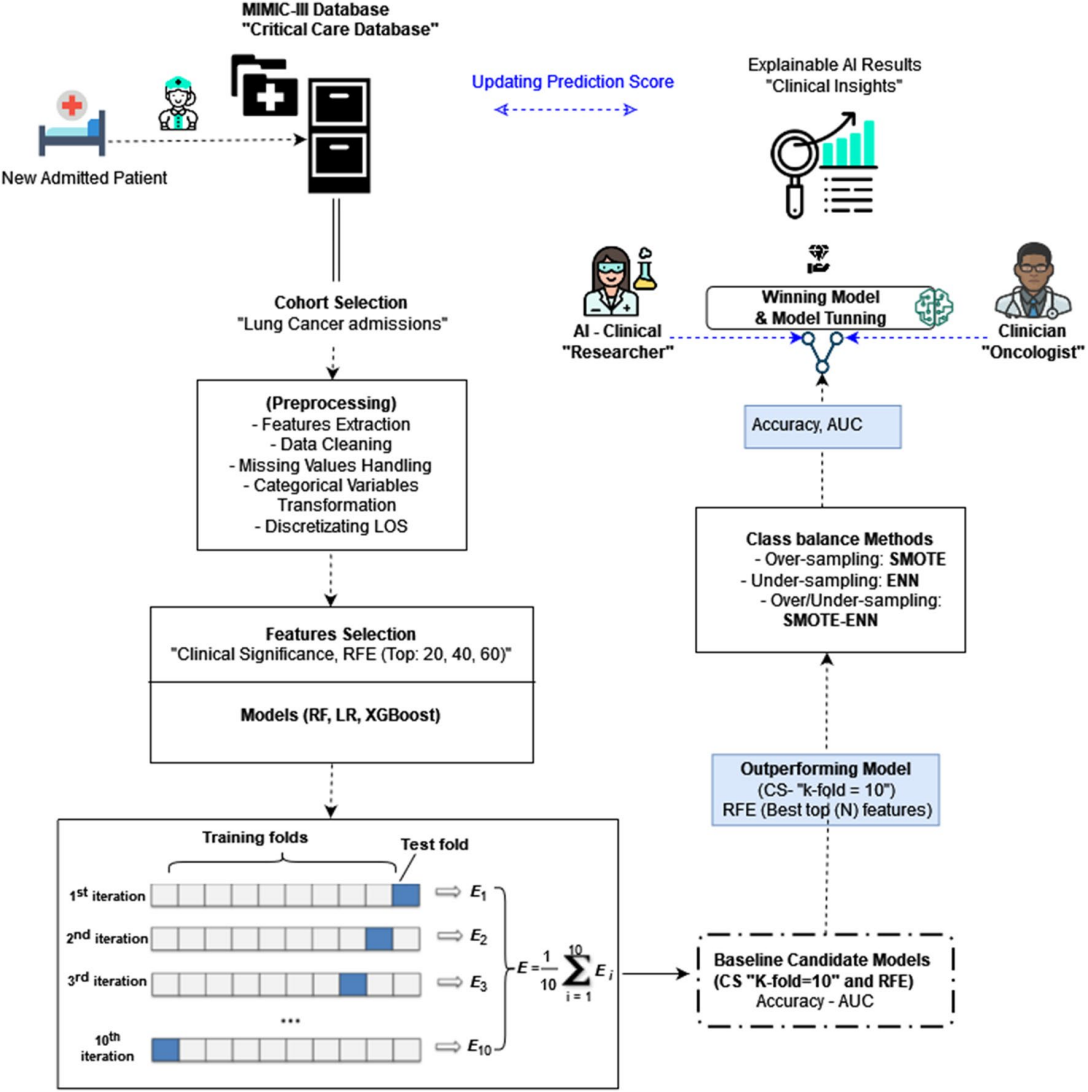
Lung cancer LOS predictive framework in ICU settings.

The feature selection procedure is substantial in feature engineering to identify and select a subset of input variables (attributes) most relevant to the target class. In this research, we have considered two feature selection techniques; the first is the clinical significance (CS) [Supplementary file: S4.4] and the second one is the Recursive Feature Elimination (RFE) [Supplementary file: S4.5]. All predictive steps in the framework for lung cancer LOS are illustrated in Fig. 3.

### Class-balancing methods

In binary class predictive tasks, one class may dominate the other class. This occurs when one class has the vast majority of the observation for the target predicted class (e.g., short LOS). This problem arises when the distribution of the classes is skewed (biased). The problem or the class distribution can be minor skewed or intemperate skewed (or imbalanced). The imbalanced data pose an extensive challenge to machine learning models, especially because most machine learning models are designed to deal with assumptions of an equal number of samples (for each class). Ignoring this problem may cause errors in the minority class (hence becoming sensitive to classification errors), leading the ML model to ignore the observation in the minority class. Therefore, treating this issue is vital to ensure the predictive model’s success, thus providing reliable results, especially in electronic medical records (EHR) domains. Our research refers to the majority class with (Short LOS), and the minority is the (Long LOS). This research employed six class balancing techniques and compared their performance in a binary class predictive task. The six class-balancing techniques are described in [Supplementary file: S5.1–S5.6].

### Models selection

The research target predictive class is binary; therefore, it is a classification problem. Suitable predictive classification algorithms are selected considering their robustness in binary prediction problems. Subsequently, the overall performance, including mean accuracy, precision, sensitivity, specificity, f1-score, IBA, G-mean and AUC scores, are evaluated. Finally, the model tuning stage is applied to the outperforming model. We have implemented three machine learning predictive models to assess the proposed Lung Cancer LOS (see Additional file: S6.1-S6.3). Some literature review studies scrutinized ensemble-based models (e.g., RF) in predicting LOS in clinical settings^15,16^. The LR is currently in used LOS, such as^14,23^ predictive problems. The XGboost model has not been examined in the literature review with LOS predictive tasks to the best of our knowledge. In this research, the RF model (Bagging), [Supplementary file: S6.1] is assessed and compared to other prominent classifiers such as XGBoost (Boosting), [Supplementary file: S6.2] and Logistic Regression [Supplementary file: S6.3]. The outperforming model to be selected as the winning model for the LOS lung Cancer framework evaluation in class-balancing and model clinical explanation. Table 3 [Supplementary file: S2.3] compares RF, XGBoost, and LR via their pros and cons.

### Models performance and evaluation

In this research, we have used a suite of evaluation metrics to evaluate the predictive models’ performance. The accuracy (mean cross-validation “k-fold” accuracy, Fig. 3), Index Balanced Accuracy (IBA), Geometric Mean Score (GMS), Precision, Sensitivity, Specificity, F1-score, and Area Under the Curve (AUC) are used in this research. In addition, we have employed a set of formulas during the three main phases of framework performance evaluation [Supplementary file: S7.1–S7.7].

### Machine learning predictions explainability with SHAP

Model explainability refers to how a human can consistently predict the model results^37^. In the machine learning domain, the higher the explainability of a certain model, the better it is for someone to understand and comprehend the predictions that have been made. We utilized the SHAP^37^ for the purpose that each SHAP value represents how much such a particular feature (independent feature) contributes to the outcomes of a specific event (predicted case). Thus, our contribution is that the SHAP is a useful machine learning explainable method that provides health clinical information systems guidance through the use of the explainable artificial intelligence (xAI) approach such as (SHAP) to make a clinical sense of the prediction of outperforming classifier.

## Conclusion and future work

Our study represents the potential of machine learning to predict the Length of Stay of ICU cancer-based hospitalization in particular lung cancer patients efficiently. We have evaluated suitable class balancing methods to deal with the imbalanced class problem, primarily challenging to the predictive modeling task because of the severely skewed class distribution in clinical health records data (clinical EHR). To the best of our knowledge, the presented LOS research framework is the first to be used for lung cancer hospitalization and predicting lung cancer patients’ future days in ICU hospitalizations. The framework provides a practical framework to be exploited by clinical oncologists, hospital bed managers, and healthcare givers as a robust predictive and explainable artificial intelligence tool for lung cancer patients in ICU settings. The Random Forest ensemble classifier has proven itself robust in different feature selection procedures (RFE or clinical significance) among the examined machine learning methods, features selection, and class balancing techniques.

Furthermore, the class balancing with Over-sampling such as ADASYN and SMOTE achieved the most outstanding AUC and G.Mean results, followed by the over/and under-sampling methods. However, Under-sampling methods did not achieve reliable results in terms of the AUC and D.mean metrics. Finally, the Random forest and the outstanding class balancing methods are explained to non-artificial intelligence experts using the SHAP machine learning explainable method.

## Supplementary Information

Supplementary Information 1.

## Data Availability

Requests for data and code should be addressed to B.A and F.A.
