# Supplementary material for: An explainable machine learning framework for lung cancer hospital length of stay prediction
Source: Sci Rep. 2022 Jan 12;12:607. doi: 10.1038/s41598-021-04608-7 (PMC8755804; doi:10.1038/s41598-021-04608-7)
Supplement: Supplementary file 1 — Supplementary Information 1. [file 41598_2021_4608_MOESM1_ESM.pdf]

S1. Research Background

Numerous statistical-based studies examined the risk factors for LOS in hospital. However, the conventional statistical models have limitations in processing multiple unprocessed variables and in their application to real healthcare data. This led scientists recently to adopt machine learning in the development of prediction models [1].

Machine learning (ML) models and healthcare analytics have recently proved to be powerful tools to recognize EHR patterns [2]. The ML predictive algorithms assume that there are data and associations and relationships between clinical variables (predictors/independents) and the target variables (dependents). Hence, ML models are capable of predicting the ICU LOS remaining time to improve hospital resource management and scheduling [3]. Recent studies have exploited machine learning algorithms to predict inpatient LOS in the context of healthcare assessment systems in clinical settings.

The non-regression ML algorithms acquired great importance to improve outcomes research. Many of these algorithms are expected to deal with a large number of variables in sophisticated and nonlinear ways, producing very efficient complex predictions [4, 5]. Based on these techniques, predictive models may assist healthcare systems in identifying clinically significant risk or identify unique and unusual risk predictors [6].

S2. Tables

S2.1 Lung Cancer LOS attributes characteristics

Table 1 Lung Cancer LOS attributes characteristics (features by group, type, mean, standard deviation (std), min value, max value, and feature type)

Feature	mean	std	min	max	Type	*P*-value
**Complete Blood Count Information**						
RBCs	3.491681	0.346278	2.49	4.6	Continuous	0.536
WBCs	11.23081	4.080906	1	22.2	Continuous	0.862
Platelets	239.9916	90.13732	23	491	Continuous	0.323
Hemoglobin	10.55518	1.127045	7.6	13.2	Continuous	0.259
Hemocrit	32.04676	3.797873	22.5	41.5	Continuous	0.613
**Differential Information**						
Bands	1.260504	1.52639	0	11	Continuous	0.100
Neutrophils	81.83613	4.391396	46	93.5		0.977
**Vitals Information**						
Temperature (F)	97.79863	1.86576	82.25	100.3	Continuous	0.983
Heart rate	91.54513	16.08313	56	155	Continuous	0.080
Respiratory rate	19.1562	3.865609	10.4	27.82353	Continuous	0.367
Systolic blood pressure	112.818	14.42322	76.82143	150.1667	Continuous	0.195
Diastolic blood pressure	59.06757	8.691874	41	78.22222	Continuous	0.885
Pulse oximetry	97.06236	1.345939	91.83333	99.69231	Continuous	0.575
**Labs Information**						
troponin	0.068109	0.02044	0.01	0.235	Continuous	0.671
BUN	19.89356	9.919206	5	81.5	Continuous	0.932
INR	14.94034	0.841213	12.6	18.5	Continuous	0.395
PTT	29.93655	4.387553	19.8	50.1	Continuous	0.515
creatinine	0.863025	0.324706	0.3	2.9	Continuous	0.902
glucose	135.0999	26.96387	74	205	Continuous	0.519
sodium	137.9699	3.94132	120	147	Continuous	0.821
potassium	4.155406	0.436217	2.9	5.15	Continuous	0.976
chloride	103.3396	4.385687	85	112	Categorial	0.263
PEEP_Set	4.892157	1.079598	0	9.333333	Continuous	0.923
tidal_volume	497.8605	67.35754	0	700	Continuous	0.174
anion_gap	13.13025	1.426835	8.5	18	Continuous	0.293

Inspired_O2_Fraction	50.67107	5.678632	30	83.33333	Continuous	0.997
Demographic Information						
GENDER	0.394958	0.490909	-	-	Binary	0.966
Admission Type						0.440
ADM_ELECTIVE	0.420168	0.495673	-	-	Categorial	
ADM_EMERGENCY	0.571429	0.496964	-	-	Categorial	
ADM_URGENT	0.008403	0.09167	-	-	Categorial	
Age Category						0.703
AGE_middle_adult	0.168067	0.375507	-	-	Categorial	
AGE_senior	0.831933	0.375507	-	-	Categorial	
Medications Information						
amiodarone	0.042017	0.201476	-	-	Binary	0.614
ampicillinsulbactam	0.033613	0.180994	-	-	Binary	0.444
atropine	0.016807	0.12909	-	-	Binary	0.592
calciumgluconate	0.210084	0.409091	-	-	Binary	0.439
carvedilol	0.008403	0.09167	-	-	Binary	0.706
cefazolin	0.235294	0.425976	-	-	Binary	0.342
cefepime	0.016807	0.12909	-	-	Binary	0.592
ceftriaxone	0.033613	0.180994	-	-	Binary	0.452
clonazepam	0.02521	0.157426	-	-	Binary	0.004**
clopidogrel	0.016807	0.12909	-	-	Binary	0.592
dextrose	0.848739	0.708767	-	-	Binary	0.014
diazepam	0.033613	0.180994	-	-	Binary	0.452
digoxin	0.016807	0.12909	-	-	Binary	0.110
diltiazem	0.042017	0.201476	-	-	Binary	0.614
diphenhydramine	0.218487	0.414967	-	-	Binary	0.398
enoxaparin	0.008403	0.09167	-	-	Binary	0.706
fentanyl	0.02521	0.157426	-	-	Binary	0.277
fentanylcitrate	0.218487	0.414967	-	-	Binary	0.253
fluconazole	0.02521	0.157426	-	-	Binary	0.277
fondaparinux	0.008403	0.09167	-	-	Binary	0.706
furosemide	0.12605	0.33331	-	-	Binary	0.080
glucagon	0.142857	0.351407	-	-	Binary	0.371
haloperidol	0.008403	0.09167	-	-	Binary	0.008**
heparin	0.739496	0.440766	-	-	Binary	0.572
hydralazine	0.042017	0.201476	-	-	Binary	0.390
hydromorphone	0.361345	0.482421	-	-	Binary	0.368
insulin	0.436975	0.498109	-	-	Binary	0.176
levofloxacin	0.10084	0.30239	-	-	Binary	0.168
levothyroxine	0.10084	0.30239	-	-	Binary	0.642
metoclopramide	0.05042	0.219736	-	-	Binary	0.761
metoprolol	0.327731	0.471371	-	-	Binary	0.528
metronidazole	0.117647	0.323552	-	-	Binary	0.294
midazolam	0.109244	0.313264	-	-	Binary	0.037
nitroglycerin	0.042017	0.201476	-	-	Binary	0.614
nitroprusside	0.008403	0.09167	-	-	Binary	0.008**
norepinephrine	0.042017	0.201476	-	-	Binary	0.614
ondansetron	0.352941	0.479905	-	-	Binary	0.686
phenytoin	0.05042	0.219736	-	-	Binary	0.118
piperacillin	0.05042	0.219736	-	-	Binary	0.118
potassium_y	0.378151	0.486976	-	-	Binary	0.853
prednisone	0.067227	0.251473	-	-	Binary	0.993
propofol	0.193277	0.396538	-	-	Binary	0.030

vancomycin	0.142857	0.351407	-	-	Binary	0.145

**. Correlation is significant at the 0.01 level (2-tailed).

The number of the attributes associated with the lung cancer patients comprised a set of (complete blood count, differential, white blood count (WBC), vital signs, laboratory tests, demographics, medications) as seen in Table 1. We extracted these variables from previous works [7, 8] for further data processing. A clinical oncologist involved in this study affirmed the attributes selection with the relevance to Lung cancer diseases. Eventually, the number of features that we inserted is 75 attributes. Other non-significant clinical variables to lung cancer LOS were dropped from the table according to the inclusion criteria (Figure *1*). These variables are ("basophils, eosinophils, polymorphonuclear leukocytes, surface culture, daily weight, lymphocytes, monocytes, triglycerides, , @0.9SodiumChloride, acetominophen, albuterol, aspirin, atenolol, epoetin, hydrochlorothiazide, ipratropium, levetiracetam, lisinopril, neostigmine, oxycodone, pantoprazole, phenylephrine, phytonadione, ranitidine, statin, trazodone, zolpidem, INS_Government, INS_Medicaid, INS_Medicare, and INS_Private).

S2.2 benefits and drawbacks of the class balancing class technique

Table 2 presents the benefits and drawbacks of

the class balancing class technique in predictive machine learning tasks

Technique	Benefits	Drawback
**SMOTE**	-Increases the feature availability to each class and prevents information loss.<br>- May overcome the issue of overfitting of datasets [9].<br>-Improved performance in low-dimensional data [10].	- Increases training time and memory to hold training data [11] (computational cost).
**ADASYN**	Enhances the learning about the distribution of the sample in a more efficient way [12]<br>- In minority and majority classes, it does not sacrifice one class in the preference for another [13].	- Risk of generating many false positives due to the generated synthetic data may be very similar to the majority class.
**ENN**	Reducing the number of training data samples and improves storage and model run time.<br>- Removes unwanted overlaps between classes [11].	Ignore useful information which might be important building rule model.
**TomekLinks**	- Treat outliers efficiently.<br>- Removes unwanted overlaps (points) between classes [9].	- low performance in the binary class prediction<br>- Many samples are removed if the decision boundary is unclear.
**SMOTE-ENN**	Good performance in small datasets [14]	Can remove more examples in-depth

	- Adjust class distribution [11].	
**SMOTE-Tomek**	Good performance in small datasets [14].	introducing artificial minority class examples too deeply in the majority class may lead to overfitting

S2.3 LOS Lung Cancer Models' Description

Table 3. LOS Lung Cancer Models' Description

Model	Model's Advantages	Model's Disadvantages
Random Forest (RF)	- Robust against overfitting in decision trees.<br>- Works well with categorical and continuous variables.<br>- Desired Predictive Performance in many contexts.	- Complexity (Creates a lot of trees) to make a final predictive decision.<br>- Computational Cost (Longer Training Time)
Xtreme Extreme Gradient Boosting (XGBoost)	- less feature engineering requires (can handle missing values and normalization).<br>- less prone to over-fitting.<br>- Speed and performance.	- difficult in interpretation.<br>- May lead to over-fitting if hyperparameters are not tuned correctly.
Logestic Regression (LR)	- Easy to implement and Fast to train.<br>- interpreted model’s coefficients as an indication for feature importance.	- Suffers from Overfitting in high-dimensional datasets.<br>- Not able to work with nonlinear problems

S3. Figures

S3.1 Inclusion Protocol for Lung Cancer Patients from MIMIC-III dataset

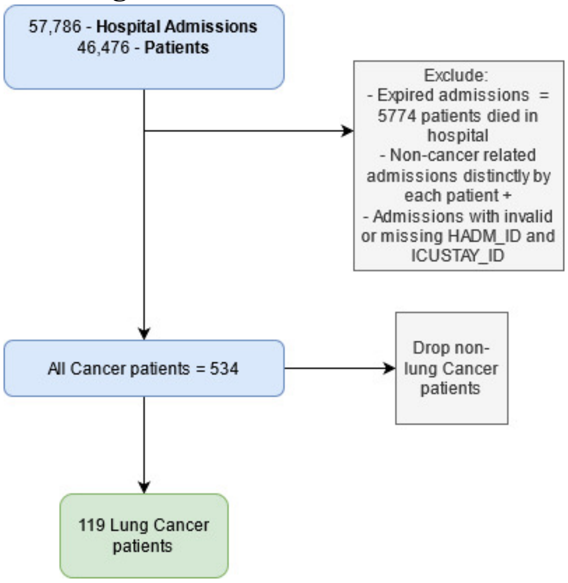

Figure 1 Inclusion Protocol for Lung Cancer Patients from MIMIC-III dataset.

The inclusion mechanism considered only ICU hospitalized patients. At the first screening, we excluded all patients who died in the hospital from the inclusion protocol. Further, we have dropped all events with missing HDAM_ID and ICUSTY_ID (a unique ID to link unique ICU stays with each HDAM_ID) from the inclusion criteria. We applied an additional inclusion criterion comprising diagnosis codes for lung cancer hospitalizations (162.x) identified by the International Classification of Diseases (ICD)9. Accordingly, we included 119 lung cancer patients in our study from the whole dataset. Figure 1 reveals the inclusion protocol for lung cancer patients in this study.

S3.2 Distribution of the patient in the Lung cancer dataset (short LOS and long LOS classes)

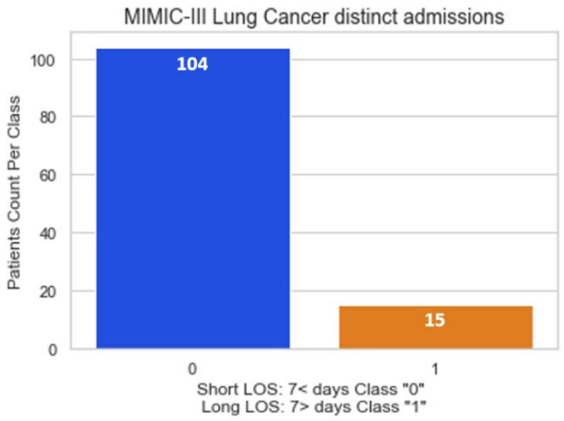

Figure 2. Showing number of patients per class (Majority-Short LOS ”0”, Minority-Short LOS ”1”)

S4. Data Prepossessing and Features Selection

S4.1 Data Imputation

We applied a null function from the Pandas library in Python [15] to verify and eliminate records with frequent missing values for each admission. This decision was coordinated with the clinical oncologist and made only in the records with cells (entries) that suffer from many missing values that cannot be

replaced by any handling missing values techniques (*Figure 3*). We disregarded any event (lung cancer admission) that lacks clinical insights to avoid any negative impact on the prediction models' performance and the overall research aim. In the case of the missing values that did not cause any absence of each (event/admission) picture, we imputed the missing values with entries based on the variable median method [7].

S4.2 Discretizating Target Class (LOS)

Discretization is the process of transferring (numeric/continuous) variables into (nominal/categorical) variables (bins). Several Artificial intelligence (AI) studies such as [16, 17] practiced continuous variables transformation into nominal/ categorical variables to be examined in various machine learning and statistical methods. Healthcare and clinical Decision support System studies [18, 19] binned the continuous variables into nominal target variables. In hospital healthcare systems, binning the (continuous) Length of Stay into nominal/ categorical is accompanied by advantages for healthcare caregivers to maximize hospital resource utilization [20, 21]. This can be achieved by binning LOS (continuous variable) into classes (labels) to help healthcare workers initially predict patients' future Stays at hospital admission.

The binning process in discretizing the LOS variable into categories (labels) is studied to facilitate LOS prediction methods. Previous research works categorized Length of Stay into different labels. For Instance, Zebin et al. [22] grouped short LOS to (0-7 days) and the long LOS to (>7 days). Similarly, Allard [23] [add more] et al. categorized Long LOS to (> 7 days) and Short LOS to below 7 days.

In this research, we binned LOS (continuous variable) into a **binary learning** LOS approach based on previous studies using the discretized scaled label "LOS" into two labels: 1) Label zero (0) for a short length of Stay or (Short LOS, 0-6 days), and label one (1) for a long length of Stay (Long LOS, 7 + days), in Figure 2.

S4.3 Categorical Variable Transformation

In this research, we implemented the (One-hot-encoding or “nominal encoding”) method [8] to transform (independent) categorical variables before building the LOS predictive models. This method aims to transform categorical to (nominal and binary) attributes that improve machine learning models' performance.

S4.4 Features selection with Clinical Significance

We disregarded the non-clinical significant variables from the inclusion criteria. The inclusion and exclusion decision for the significant clinical variables was affirmed with the clinical oncologist. This decision was necessary for the study. Firstly, to ensure all clinical features of Lung Cancer are considered and the non-important features are eliminated. Therefore, the feature selection (CS) puts the patients on the Length of stay prediction perception from a clinical perspective. Secondly, it helped to reduce the features' dimensionality' and improve machine learning models' performance in the baselining stage. The disadvantage of the approach is that it may leave weak associations between independent and dependent variables and impact the performance's predictive models. AI-based cancer studies [24, 25] utilized features' selection with the CS approach in machine learning predictive tasks. Table 1 shows variables selection with a clinical significance approach.

S4.5 Features Selection with Recursive Feature Elimination (RFE)

The principle of the RFE technique [26] is based on selecting features recursively. This is achieved by removing a smaller set of attributes per loop. This process occurs recursively, and the weakest features are eliminated at the end. The features are ranked by the model's coefficient (coef) or feature importance. The optimal set of features are attained using cross-validation. RFE has been utilized in cancer-based studies such as in [27-29]. RFE is achieved using the algorithm (Figure 3)

S5. Proposed Algorithms in the study

S5.1 SMOTE

The Synthetic Minority Oversampling Technique (SMOTE) is an oversampling technique applied in imbalanced datasets in classification problems. The SMOTE is an over-sampling method in which the minority class involves creating synthetic elements of minority class examples based on the existing ones. It picks up a point from the minority class and calculates the nearest neighbors by the Euclidean distance between data points in the feature space.

S5.2 ADASYN

The Adaptive Synthetic (ADASYN) algorithm [13] is an oversampling method similar to the SMOTE technique. It works by generating many samples for a given feature vector (x_i). The (x_i) is proportional to a number of the nearby samples, and it does not belong to the same class as (x_i). This helps to deal with outliers, especially when generating new synthetic training examples.

S5.3 Edited Nearest Neighbors (ENN)

The Under-sampling with Edited Nearest Neighbors (ENN) method applies the nearest-neighbors algorithm. It edits the dataset and removes the samples from the dataset that do not agree enough with their neighborhood [30]. In ENN, the nearest-neighbours are computed, and if the selection criterion is not satisfied, the sample is removed. This process or (removing noise from samples) ensures each sample in a class to be under-sampled.

S5.4 Tomek Links

Tomek links algorithm [31] is an under-sampling method that detects a pair of observations (two different samples from different classes x and y) near to teach others. The pairs are called Tomek link. The Tomek link is defined for any sample (*z*):

S5.5 SMOTE+ENN

The SMOTE+ENN algorithm [14] is a hybridization technique (combination of over-and under-sampling) where SMOTE helps to do extensive data cleaning. Further, the misclassification caused by NN (nearest-neighbors) samples is removed [32] in both classes (Short LOS and Long LOS). This results in achieving a clear, concise and separation between classes.

S5.6 SMOTE-Tomek

The SMOTE+Tomek algorithm [14] is a hybridization technique combination of over-and under-sampling) that combines the oversampling (SMOTE) and the undersampling (Tomek-Links) techniques in order to achieve optimized performance for the classifier. The SMOTE+Tomek first applies SMOTE to the minority class (e.g., Long LOS) to a balanced distribution, then the examples from the majority class (Short LOS) in Tomek Links are identified and removed.

S5.7 Pseudocode for LOS Lung Cancer Framework

```
Pre-processing data
Input: load extracted dataset MIMIC-III (d^e):
Output: cleaned & processed dataset (d^p)
Function (CTM):
   For each row R in d^e:
         If R in d^e contains:
           -invalid & expired admissions
           -missing records > threshold value (th_v)
           -non-cancer events (e)
         end if
   End For
dataset (d^c ) ← Return and save R in the cleaned dataset
```

```
Data Discretization and Transformation
Input: load dataset (d^c )
Output: Discretized dependent (y: LOS) – Transformed set of independents features x= [x1, x2, . . .xn]
Function (DLOS):
    For each row Ry in y:
        If y≥0 & <cut_point:
            Label Rr = 0
        else:
            Label Rr = 1
        end if else
    end For
Function (TLOS):
    While (F is not Continuous):
        For each Categorical Feature F in d^c:
            Transpose F← f = (f1, f2, f3, . . . , fn)^T
        end For
    Break while
Features selection with Recursive Feature Elimination (RFE)
Inputs:
    → dataset d^c
    → Set of x features F= [x1, x2, . . .xn]
    → Ranking model M(D, F)
Procedure:
    "Recursive loop"
    For i in [1 to x]:
            Rank F with M(D, F)
            f* ← last ranked feature in F
            R (x – i +1) ← f*
            F ← F - f*
Output:
Optimal Ranked Features (R)
where:
CTM is the function to clean & treat Missing
DLOS is the function to discretize the continuous LOS
TLOS is the function to transform categorical features
(thv) is the threshold of missing values in each R= 70%
cut_point: the cut point of 7+ days
Fis a categorical feature with (f1, f2, f3, . . . , fn) layered f distinct values
```

Figure 3 Pseudocode for LOS Lung Cancer Framework

S5.8 Pseudocode for the six class balancing methods

Input: dataset (D); majority sample (S_{maj}) "S-LOS", and minority sample (S_{min}) "L-LOS"
Output: balanced dataset

Oversampling

SMOTE (X_{new}) steps:

1. Identify feature vector (x_i) and its nearest neighbor's (x_{zi}), where u is a number, and it is randomly chosen from U (0,1).
2. Take the two difference between (x_{zi}) and (x_i).
3. Multiply the difference ($x_{zi} - x_i$) with Random number (u) between (0, 1).

4. Identify a new point on the line segment by Adding the random to feature vector
5. Repeat the process for the identified features.
6. The new generated SMOTE synthetic example is obtained as follows:

$$X_{new} = x_i + u * (x_{zi} - x_i)$$

ADASYN (S_i) steps:

1. Calculate the ratio of majority to minority (d $= \frac{m_s}{m_l}$). where d ϵ (0,1], (m_s) is the minority class and (m_l) is the majority class.
2. Initiate the algorithm when (d< d_{th}), where d is lower than a certain threshold (d_{th}).
3. Calculate the total number of the synthetic data sample to generate the minority class (G) to generate:

   $$G = (m_l - m_s) * \beta$$

   where λ ϵ (0,1] (random number) and $(x_{zi} - x_i)$ is the difference in *n* denominational space.
4. Find *K* (nearest neighbors) for each x_i ϵ *minority_class* according to Euclidean distance in *n* denominational space, then calculate the ratio (r_i):

   $$r_i = \frac{\Delta_i}{K}$$

   where (Δ_i) is the number of samples in the *K* nearest neighbors of x_i that belongs to the majority class. r_i ϵ (0,1]
5. Normalize (r_i) values in which the sum of all r_i values are equal to:

   $$r_i = \frac{r_i}{\sum r_i}, \sum \hat{r}_i = 1$$

   where $\hat{r}_i$ is the density distribution.
6. Calculates the number *G* of synthetic data examples that are required to be generated for each minority example x_i.

   $$g_i = \hat{r}_i * G$$

7. For each minority class data sample (x_i) for each neighborhood, generate g_i synthetic data sample;
   1) Randomly choose one minority data sample (example) within the neighborhood (x_{zi}).
   2) the new generated synthetic example is achieved with the following equation (ADASYN):

   $$S_i = x_i + (x_{zi} - x_i) * \lambda$$

Undersampling

ENN (*N*) steps:

1. Obtain k nearset neighbour of x_i, x_i ϵ N
2. Remove x_i if number of neighbours from another class is dominant.
3. Repeat process for every majority instance of subset N

Tomek Link for sample (*z*) steps:

1. Tomek links identification
   d(x,y) < d(x,z) and *d(x,y) < d(y,z)*
   where *d(.)* is the distance between the two samples
2. Borderline and noise examples removal between the two samples

Over/Under-sampling

SMOTE-ENN steps:

1. Over-sampling using SMOTE
2. cleaning using ENN

SMOTE-Tomek steps:

1. Over-sampling using SMOTE
2. cleaning using Tomeklink

Figure 4 Pseudocode for the six class balancing methods

S6. Machine Learning Models

S6.1 Random Forest

Random Forest (RF) algorithm [33] is an ensemble learning model and classification-based method. The RF model works by generating Random subsets from the original dataset (bootstrapping). Then, in each node in the decision tree, only a random set of features are to be considered for deciding the best split. After that, a decision tree model is fitted on each (of the subsets). The final output (prediction) is achieved by calculating the average predictions from all decision trees. To summarise, the model operates by randomly selecting data points and features and then building multiple trees (forests).

The RF classifier was appropriated in this study for the LOS lung cancer predictive framework with Gini Index (IG [34]) is implemented:

$$I_G = 1 - \sum_{i=1}^{C} (P_i)^2 \qquad (1)$$

Where *Pi* proportion of samples that belongs to a class (C) for a particular node.

S6.2 XGBoost

The eXtreme Gradient Boosting (XGBoost) algorithm [35] is an ensemble-based learning (bagging) model. The XGBoost is an implementation of the gradient boosted decision trees [36] designed for performance and speed. It uses more regularized model formalization to control the over-fitting, giving it better performance [35].

Considering dataset (d^c) with m features and n of examples, where $d^c=\{(x_i,y_i)\}$ ($x_i \in R^m$, $y_i \in R$, i = 1,2, . . .,n), the XGBoost model can be described as the following [37]:

$$\hat{y}_i = \sum_{k=1}^{K} f_k(x_i), \quad f_k \in F(i = 1,2, \dots, n) \qquad (2)$$

That $F = \{f(x) = w_{q(x)}\}(q: R^m \rightarrow \{1,2, \dots T\}, w \in R^T)$ is the CART decision tree structure set, *q* is the tree structure of the sample map to the leaf nodes, *T* is the number of leaf nodes, and *w* is the real score of leaf nodes.

When constructing the XGBoost model, finding the optimizer is necessary to establish an optimal model. Therefore, the objective function of the XGBoost is divided into an error function *L* term, and a model complexity function Ω. Then the objective function is written as the following:

$$obj = L + \Omega \qquad (3)$$

$$L = \sum_{i=1}^{n} (y_i - \hat{y}_i)^2 \qquad (4)$$

$$\Omega = \gamma T + \frac{1}{2}\lambda \sum_{j=1}^{T} w_j^2 \qquad (5)$$

where γT is the regular term of *L1*, $\frac{1}{2}\lambda \sum_{j=1}^{T} w_j^2$, is the regular term of *L2.* γ and λ are adjustment parameters to prevent the model from overfitting.

Now the objective function is expressed as:

$$Obj^{(t)} = \sum_{i=1}^{n} \left(y_i - \left(\hat{y}_i^{(t-1)} + f_t(x_i) \right) \right)^2 + \Omega \qquad (6)$$

where $\hat{y}_i^{(t-1)}$ is the predicted value of t-1th model and $f_t(x_i)$ is the new function added at *t*th time. The *Obj* is a scoring function that is used as an evaluation model; noting that the smaller the *Obj* value, the better the model affect.

S6.3 Logistic Regression

The logistic regression (LR) [38, 39] is a statistical model that uses the logistic function to predict dependent variables from the independents used. It is used in machine learning in predictive binary tasks (classification). The logistic function is formulated as the following:

$$logistic(n) = \frac{1}{1+\exp(-n)} \quad (7)$$

S7. Performance evaluation metrics

S7.1 Accuracy:

Donates the ratio of the correct predictions to the total of a number of predictions.

$$Accuracy = \frac{TP+TN}{TP+TN+FP+FN} \quad (8)$$

Where, TP: True Positive, FP: False Positive, FN: False Negative, TN: True Negative.

The accuracy does not describe the predictive story in imbalance-class datasets. Therefore, other metrics are used to evaluate imbalanced data, such as precision, Sensitivity, G.mean and IBA.

S7.2 Precision:

Refers to the number of positive classifications that are actually correct (or called positive predicted value '**PPR**').

$$Precision = \frac{TP}{TP+FP} \quad (9)$$

S7.3 Sensitivity (Recall):

Measures the proportion of actual positives that is well classified (or called the true positive rate '**TPR**' or Recall).

$$Sensitivity = \frac{TP}{TP+FN} \quad (10)$$

S7.4 Specificity:

Measures the proportion of actual negatives that is well classified (true negative rate '**TNR**')

S.7.5 F1-Score:

It can be interpreted as the weighted average of precision and recall. F1-Score = 1 is the best possible value, and F1-Score close to 0 is the worst value.

$$F1-Score = 2 * \frac{\boldsymbol{Preision * Recall}}{\boldsymbol{Precision + Recall}} \quad (11)$$

S7.6 Index Balanced Accuracy (IBA):

The Generalized IBA [40] is used to weigh a suitable measure to evaluate an imbalanced datasets' performance. The weight factor assists in favoring those results with the better classification rates on the minority class. Formula

$$IBA_\alpha(M) = (1 + \alpha.Dom).M \quad (12)$$

where *Dom* is the domenance; *Dom* = *TPR* – *TNR* within the range [-1, +1]

Dom is used to estimating the relation between TPR and TNR. The closer Dom to (0), the more balance with both individual rates are achieved.

and it is weighted by $\alpha \geq 0$ to reduce its influence on the results of the particular metric M

$1 + \alpha.Dom$ is the weighting factor

S7.7 Area Under the ROC Curve (AUC)

The AUC measures the quality of the model's predictions regardless of what classification threshold is chosen. It represents the area under the ROC curve plots (TPR vs. FPR)

$$\text{TRP} = \frac{TP}{TP+FN}, \qquad \text{FPR} = \frac{FP}{FP+TN} \tag{13}$$

where TPR is the true positive rate, and FPR is the false positive rate.

S7.8 Geometric Mean Score (G.Mean):

The G.mean [41] aims to maximize each of the classes' accuracy while keeping the accuracy balanced.

$$G.mean = \sqrt{TPR * TNR} \tag{14}$$

This study refers to accuracy in the models benchmarking (baselining) performance evaluation, whereas, Precision, Sensitivity, Specificity, AUC, IBA, and G.mean during class-balancing performance evaluation.

S8. Results

S8.1 Experiments Setup

We conducted the methods evaluation and implementation on a computer with (Intel core i7), CPU speed at 1.90GHZ, and 16 GB RAM. We used Python 3 for machine learning and all framework steps development and deployment.

S8.2 Baseline Stage with Cross-Validation

The first phase is the benchmarking stage (models-baselining) with cross-validation (k-fold=10). In this phase, we assessed the three proposed predictive models (RF, XGBoost, and LR) on feature selection methods (CS) and the RFE with three varieties (Top 20 features, Top 40 features, and Top 60 features). The outcome of the first phase is the model (mean accuracy => 85%). The second phase incorporates evaluating the performance of the candidate model using six class balancing methods. The research framework integrates all these phases in the pipeline and fits the outperforming model for further clinical interpretations using the SHAP machine learning explainability (third phase).

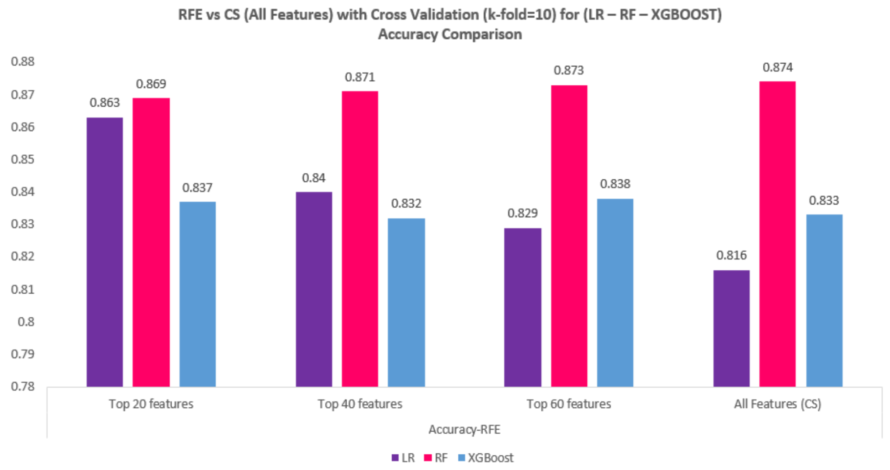

Figure 5 An Accuracy Comparison between RFE and CS (All Features) with Cross-Validation (k-fold=10) for (LR – RF – XGBoost)

A total of 119 unique lung cancer patients met the inclusion criteria (Figure 1) of our study sample. Therefore, we verified the cohort selection on the proposed models (RF, XGBoost, and LR) using cross-validation (K-fold=10), (Test is 30%) with the mean accuracy comparison and standard deviation (std) error in the mean performance as the performance evaluation metrics. We report the baselining analysis results in (Figure 5) and (Figure 6). As seen from (Figure 5), the RF achieved the best predictive results with (k-fold =10, mean accuracy 87.4%) by ensuing the CS feature selection procedure. Moreover, RF classifier attained with RFE (Top 60 features) the highest mean accuracy (87.3%) amongst the RFE model-based Top features selection procedure.

The XGBoost classifier showed a minor fluctuation, with mean accuracy ranging from 82.3% to 82.8% using two different feature selection methods (RFE and CS). In contrast, the logistic regression classifier's mean accuracy performance retreated as more features were build up in each RFE evaluation with (k-fold = 10) metric. This trend was affirmed within all features (CS), where the LR achieved the lowest mean accuracy (81.6%).

The std error in the mean performance for RF remained stable (9.9%) with RFE top features (40, 60) and all features (CS), respectively. However, the RFE (Top 20 features) evaluation had the least (std) error in the mean performance (9.7%). XGboost classifier showed an improved trend in the reported (std) error in the mean performance. While it recorded 10% of the std error in RFE (Top 40 features), it achieved an optimized (std = 9.6%) in the CS feature selection procedure with (k-fold = 10). LR classifier acquired relatively higher std error in the mean performance compared to other RF and XGboost.

While LR is the fastest model to train, the XGboost and RF needed more time to report their cross-validation results (k-fold=10) (Figure 7). Hence, RF and XGboost are more computational costly models according to the data input and number of features in this study. In analyzing reported results during the baseline phase, RF is designated for further detailed analysis in the next phase (class-balancing) performance evaluation.

S8.3 Baseline Stage with Cross-Validation

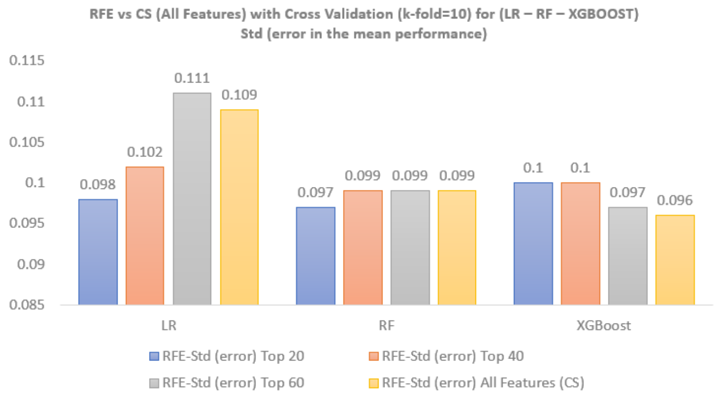

Figure 6. RFE vs CS (All Features) with Cross-Validation (k-fold=10) for (LR – RF – XGboost)

Std (error) in the mean performance)

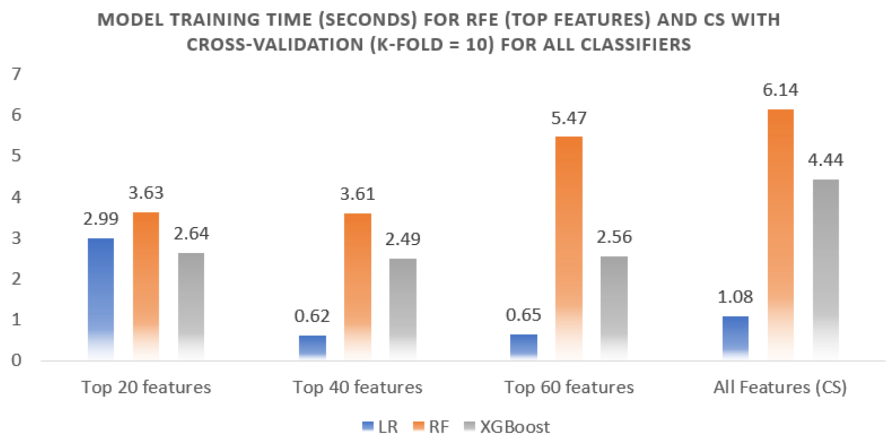

Figure 7 Time to execute Model with Cross-Validation (K-fold = 10)

S8.4 Class Balancing Results

Table 4 demonstrates the lung cancer dataset before applying imbalanced data (WCB) and utilizing various class balancing techniques. The second column in the table illustrates the short LOS percentage to the long LOS per each class-balancing approach. We foresee a better predictive performance to be achieved from the balanced data. Specific metrics (*section S7*) were being used during classification performed on balanced dataset evaluation measures.

Table 4 Percentage of Short LOS to Long LOS in the Lung Cancer dataset, benefit, and drawback of each method

**Approach for LOS Predictive Models Evaluation**	**Percentage of Short LOS to Long LOS per approach**
**Without class balancing (WCB)**	87.4% - 12.6%
**Over-Sampling**	
**SMOTE**	50% - 50%
**ADASYN**	49.66% - 50.34%
**Under-Sampling**	
**ENN**	88.1 % - 11.9 %
**TomekLinks**	89.88% - 10.12%
**Combination of Over-and Under-Sampling**	
**SMOTE-ENN**	46.3% – 53.7 %
**SMOTETomek**	50%-50%

S8.4.1 A comparison between Class-balancing Methods using Features Selection (RFE=60) method and Random Forest Model

Table 5 A comparison between Class-balancing Methods using Features Selection (RFE=60) method and Random Forest Model.

**Method**	**Precision***	**Sensitivity***	**Specificity***	**F1-Score***	**AUC***	**IBA***	**G.mean***
**Oversampling**							
SMOTE-CS	98% [95.3-100]	98% [95.3-100]	98% [95.3-100]	98% [95.3-100]	98% [95.3-100]	96% [92.2-99.8]	98% [95.3-100]
**ADASYN-CS**	100%	100%	100%	100%	100%	100%	100%

**Undersampling**							
ENN-CS	80% [72.2-87.8]	89% [82.9-95.1]	11% [4.9-17.1]	85% [78-92]	50% [40.2-59.8]	0%	0%
TomekLinks-CS	92% [86.7-97.3]	96% [92.2-99.8]	4% [0.2-7.8]	94% [89.3]	50% [40.2]	0%	0%
**Combination of over-and under-sampling**							
SMOTETomek-CS	98% [95.3-100]	98% [95.3-100]	98% [95.3-100]	98% [95.3-100]	98% [95.3-100]	96% [95.3-100]	98% [95.3-100]
SMOTE-ENN-CS	97% [93.7-100]	97% [93.7-100]	98% [95.3-100]	97% [93.7-100]	97% [93.7-100]	94% [89.3-98.7]	97% [93.7-100]

S8.5 Class-Balancing with (SHAP)

The Random Forest prediction outcomes with the four class balancing methods (Figure 8) were unlocked and explained using the SHapley Additive exPlanations (SHAP) [42]. The SHAP works by explaining the prediction of instance (x) by computing each feature's contribution to the prediction. It is also referred as a method to explain individual predictions. A TreeExplainer function [43] using the TreeSHAP [42] algorithm was exploited to visualize and explain the Random Forest (ensemble) tree model's output.

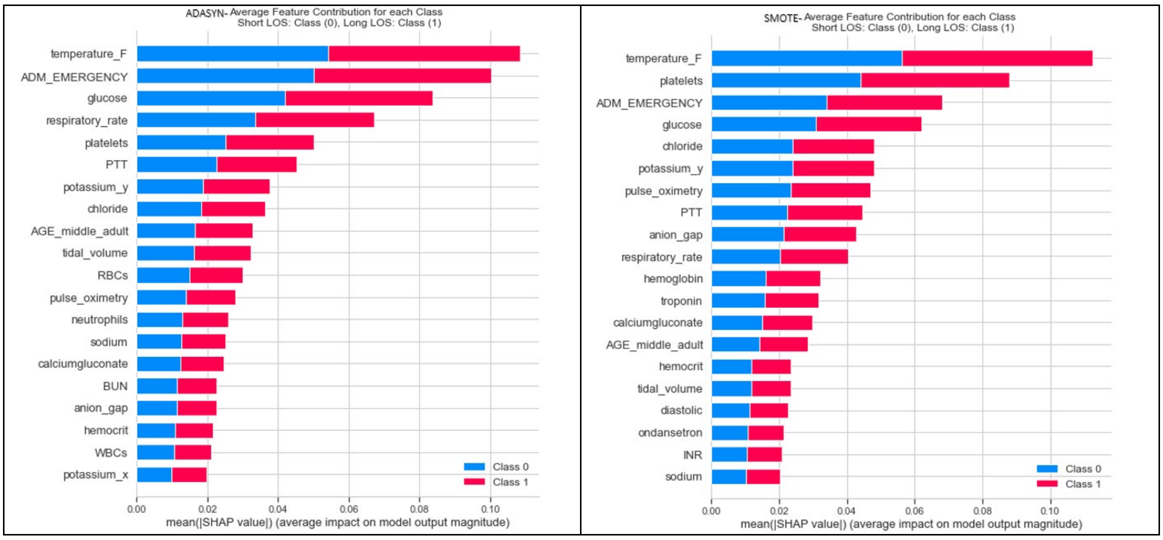

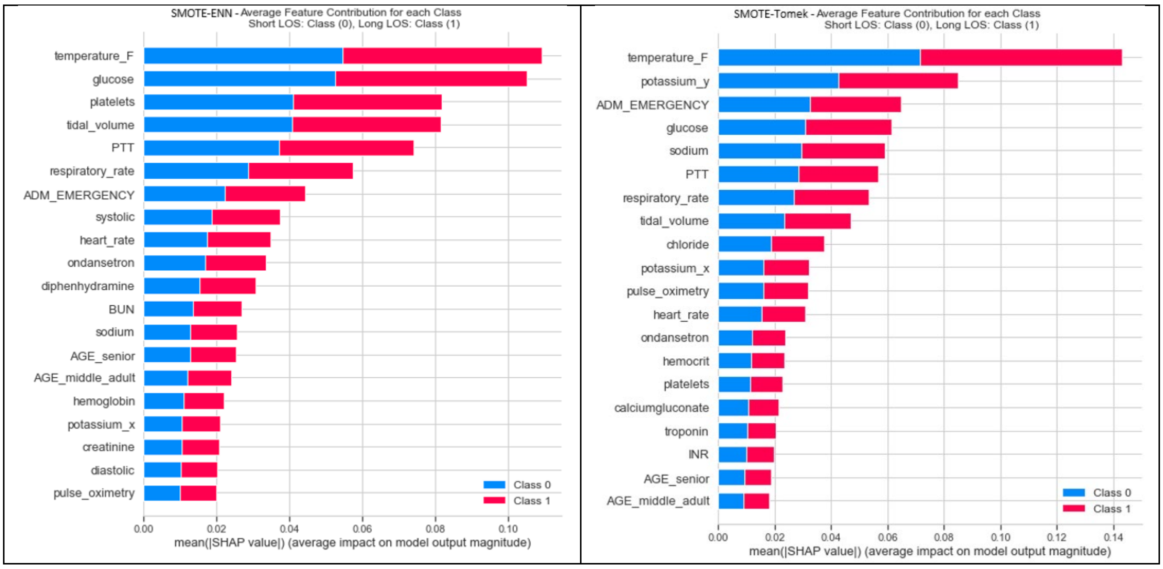

Figure 8. SHAP (mean value; the impact of each model's (features) on the model output magnitude for selected Class-balancing methods with RF.

The Random Fores's SHAP interpretations for the dependable class balancing algorithms (SMOTE, ADASYN, SMOTE-ENN, and SMOTETomek) are depicted in (Figure 8). Top features such Tempreature' tempreature_f', Emergency Admission' ADM_EMERGENCY', Glucose, respiratory rate (respiratory_rate)) were highly interpreted and are the most highly ranked features. This confirms how the SHAP using RF could rank clinical variables based on features' importance with clinical soundness. While RF-SHAP (SMOTE-ENN) ranked (systolic) variable in the top features, the diastolic came in the least in features by importance in the list.

References

[1] Y.-Y. Jo *et al.*, "Prediction of Prolonged Length of Hospital Stay After Cancer Surgery Using Machine Learning on Electronic Health Records: Retrospective Cross-sectional Study," *JMIR medical informatics,* vol. 9, no. 2, p. e23147, 2021.

[2] W. E. Muhlestein, D. S. Akagi, J. M. Davies, and L. B. Chambless, "Predicting Inpatient Length of Stay After Brain Tumor Surgery: Developing Machine Learning Ensembles to Improve Predictive Performance," *Neurosurgery,* 2018.

[3] H. Harutyunyan, H. Khachatrian, D. C. Kale, G. Ver Steeg, and A. Galstyan, "Multitask learning and benchmarking with clinical time series data," *Scientific data,* vol. 6, no. 1, p. 96, 2019.

[4] J.-E. Bibault, P. Giraud, and A. Burgun, "Big data and machine learning in radiation oncology: state of the art and future prospects," *Cancer letters,* vol. 382, no. 1, pp. 110-117, 2016.

[5] Z. Obermeyer and E. J. Emanuel, "Predicting the future—big data, machine learning, and clinical medicine," *The New England journal of medicine,* vol. 375, no. 13, p. 1216, 2016.

[6] A. G. Singal *et al.*, "Machine learning algorithms outperform conventional regression models in predicting development of hepatocellular carcinoma," *The American journal of gastroenterology,* vol. 108, no. 11, p. 1723, 2013.

[7] K. T. Baghaei and S. Rahimi, "Sepsis Prediction: An Attention-Based Interpretable Approach," in *2019 IEEE International Conference on Fuzzy Systems (FUZZ-IEEE)*, 2019: IEEE, pp. 1-6.

[8] B. Alsinglawi *et al.*, "Predicting Length of Stay for Cardiovascular Hospitalizations in the Intensive Care Unit: Machine Learning Approach," in *2020 42nd Annual International Conference of the IEEE Engineering in Medicine & Biology Society (EMBC)*, 2020: IEEE, pp. 5442-5445.

[9] V. Nagarajan, "A critical analysis of Sampling Techniques for imbalanced data classification: An application to Social Media," Dublin, National College of Ireland, 2017.

[10] L. Lusa, "Improved shrunken centroid classifiers for high-dimensional class-imbalanced data," *BMC bioinformatics,* vol. 14, no. 1, pp. 1-13, 2013.
[11] T. Le, M. T. Vo, B. Vo, M. Y. Lee, and S. W. Baik, "A hybrid approach using oversampling technique and cost-sensitive learning for bankruptcy prediction," *Complexity,* vol. 2019, 2019.
[12] A. Amin *et al.*, "Comparing oversampling techniques to handle the class imbalance problem: A customer churn prediction case study," *IEEE Access,* vol. 4, pp. 7940-7957, 2016.
[13] H. He, Y. Bai, E. A. Garcia, and S. Li, "ADASYN: Adaptive synthetic sampling approach for imbalanced learning," in *2008 IEEE international joint conference on neural networks (IEEE world congress on computational intelligence)*, 2008: IEEE, pp. 1322-1328.
[14] G. E. Batista, R. C. Prati, and M. C. Monard, "A study of the behavior of several methods for balancing machine learning training data," *ACM SIGKDD explorations newsletter,* vol. 6, no. 1, pp. 20-29, 2004.
[15] W. McKinney, *Python for data analysis: Data wrangling with Pandas, NumPy, and IPython.* " O'Reilly Media, Inc.", 2012.
[16] K. Ho and P. Scott, "Zeta: a global method for discretization of cotitinuous variables," in *Proceedings of the 3rd International Conference on Knowledge Discovery and Data Mining*, 1997, pp. 191-194.
[17] E. J. Clarke and B. A. Barton, "Entropy and MDL discretization of continuous variables for Bayesian belief networks," *International Journal of Intelligent Systems,* vol. 15, no. 1, pp. 61-92, 2000.
[18] S. Jung, Y. Bi, and R. V. Davuluri, "Evaluation of data discretization methods to derive platform independent isoform expression signatures for multi-class tumor subtyping," *BMC genomics,* vol. 16, no. 11, pp. 1-10, 2015.
[19] A. Gupta, T. Liu, and S. Shepherd, "Clinical decision support system to assess the risk of sepsis using tree augmented Bayesian networks and electronic medical record data," *Health informatics journal,* vol. 26, no. 2, pp. 841-861, 2020.
[20] A. Tabaie, F. H. Chokshi, A. L. Holder, and S. N. Nemati, "Doubly-robust estimation of effect of imaging resource utilization on discharge decisions in emergency departments," in *2018 40th Annual International Conference of the IEEE Engineering in Medicine and Biology Society (EMBC)*, 2018: IEEE, pp. 3256-3259.
[21] Y.-S. Chen, C.-H. Cheng, C.-J. Lai, C.-Y. Hsu, and H.-J. Syu, "Identifying patients in target customer segments using a two-stage clustering-classification approach: A hospital-based assessment," *Computers in Biology and Medicine,* vol. 42, no. 2, pp. 213-221, 2012.
[22] T. Zebin, S. Rezvy, and T. J. Chaussalet, "A deep learning approach for length of stay prediction in clinical settings from medical records," in *2019 IEEE Conference on Computational Intelligence in Bioinformatics and Computational Biology (CIBCB)*, 2019: IEEE, pp. 1-5.
[23] J. P. Allard *et al.*, "Decline in nutritional status is associated with prolonged length of stay in hospitalized patients admitted for 7 days or more: A prospective cohort study," *Clinical nutrition,* vol. 35, no. 1, pp. 144-152, 2016.
[24] Z. Rustam and N. Maghfirah, "Correlated based SVM-RFE as feature selection for cancer classification using microarray databases," in *AIP Conference Proceedings*, 2018, vol. 2023, no. 1: AIP Publishing LLC, p. 020235.
[25] X. Min *et al.*, "Multi-parametric MRI-based radiomics signature for discriminating between clinically significant and insignificant prostate cancer: Cross-validation of a machine learning method," *European journal of radiology,* vol. 115, pp. 16-21, 2019.
[26] P. M. Granitto, C. Furlanello, F. Biasioli, and F. Gasperi, "Recursive feature elimination with random forest for PTR-MS analysis of agroindustrial products," *Chemometrics and intelligent laboratory systems,* vol. 83, no. 2, pp. 83-90, 2006.

[27] A. Bustamam, A. Bachtiar, and D. Sarwinda, "Selecting features subsets based on support vector machine-recursive features elimination and One Dimensional-Naïve Bayes classifier using support vector machines for classification of prostate and breast cancer," *Procedia Computer Science,* vol. 157, pp. 450-458, 2019.

[28] S. Karthik, R. S. Perumal, and P. C. Mouli, "Breast cancer classification using deep neural networks," in *Knowledge computing and its applications*: Springer, 2018, pp. 227-241.

[29] S. F. Abdoh, M. A. Rizka, and F. A. Maghraby, "Cervical cancer diagnosis using random forest classifier with SMOTE and feature reduction techniques," *IEEE Access,* vol. 6, pp. 59475-59485, 2018.

[30] D. L. Wilson, "Asymptotic properties of nearest neighbor rules using edited data," *IEEE Transactions on Systems, Man, and Cybernetics,* no. 3, pp. 408-421, 1972.

[31] I. Tomek, "Two modifications of CNN," 1976.

[32] M. S. Santos, J. P. Soares, P. H. Abreu, H. Araujo, and J. Santos, "Cross-validation for imbalanced datasets: Avoiding overoptimistic and overfitting approaches [research frontier]," *ieee ComputatioNal iNtelligeNCe magaziNe,* vol. 13, no. 4, pp. 59-76, 2018.

[33] A. Liaw and M. Wiener, "Classification and regression by randomForest," *R news,* vol. 2, no. 3, pp. 18-22, 2002.

[34] M. Pal, "Random forest classifier for remote sensing classification," *International journal of remote sensing,* vol. 26, no. 1, pp. 217-222, 2005.

[35] T. Chen and C. Guestrin, "Xgboost: A scalable tree boosting system," in *Proceedings of the 22nd acm sigkdd international conference on knowledge discovery and data mining*, 2016, pp. 785-794.

[36] A. Natekin and A. Knoll, "Gradient boosting machines, a tutorial," *Frontiers in neurorobotics,* vol. 7, p. 21, 2013.

[37] S. Li and X. Zhang, "Research on orthopedic auxiliary classification and prediction model based on XGBoost algorithm," *Neural Computing and Applications,* pp. 1-9, 2019.

[38] B. Alsinglawi and O. Mubin, "Predictive Analytics and Deep Learning Techniques in Electronic Medical Records: Recent Advancements and Future Direction," in *Workshops of the International Conference on Advanced Information Networking and Applications*, 2019: Springer, pp. 907-914.

[39] S. Dreiseitl and L. Ohno-Machado, "Logistic regression and artificial neural network classification models: a methodology review," *Journal of biomedical informatics,* vol. 35, no. 5-6, pp. 352-359, 2002.

[40] V. García, R. A. Mollineda, and J. S. Sánchez, "Theoretical analysis of a performance measure for imbalanced data," in *2010 20th International Conference on Pattern Recognition*, 2010: IEEE, pp. 617-620.

[41] V. García, J. S. Sánchez, and R. A. Mollineda, "On the effectiveness of preprocessing methods when dealing with different levels of class imbalance," *Knowledge-Based Systems,* vol. 25, no. 1, pp. 13-21, 2012.

[42] S. Lundberg and S.-I. Lee, "A unified approach to interpreting model predictions," *arXiv preprint arXiv:1705.07874,* 2017.

[43] SHAP. "TreeExplainer." https://shap-lrjball.readthedocs.io/en/docs_update/generated/shap.TreeExplainer.html (accessed 15/1/2021, 2021).
